# Authors’ Reply to “Comment on: Effects of the FIFA 11+ Program on Physical Fitness in Youth and Adult Soccer Players: A Systematic Review and Meta-analysis”

**DOI:** 10.1007/s40279-026-02488-3

**Published:** 2026-07-18

**Authors:** Ibnu Noufal Kambitta Valappil, Karuppasamy Govindasamy, Gavoutamane Vasanthi, Masilamani Elayaraja, Cain C. T. Clark, Koulla Parpa, Borko Katanic, Hüseyin Şahin Uysal, Hassane Zouhal, Urs Granacher

**Affiliations:** 1 Department of Physical Education and Sports, Pondicherry University, Puducherry, 605014 India; 2 Department of Sports, Recreation and Wellness, Symbiosis International (Deemed University), Hyderabad Campus, Modallaguda (V), Nandigama (M), Rangareddy, Telangana 509217 India; 3 College of Life Sciences, Birmingham City University, Birmingham, B15 3TN UK; 4 Faculty of Sport and Exercise Science, UCLan University of Cyprus, 7080 Pyla, Cyprus; 5 Montenergin Sports Academy, Podgorica, Montenegro; 6 Department of Coaching Education, Faculty of Sport Sciences, Burdur Mehmet Akif Ersoy University, 15030 Burdur, Turkey; 7 Sport & Human Performance Research Centre, University of Limerick, Limerick, Ireland; 8 Institut International des Sciences du Sport (2I2S), 35000 Rennes, France; 9 Department of Sport and Sport Science, Exercise and Human Movement Science, University of Freiburg, 79102 Freiburg, Germany

Dear Editors,

We thank Dr. Thapa, Mr. Bruno and Dr. García-Carrillo for their interest in our recent systematic review and meta-analysis [1]. We have reviewed their commentary [2] carefully and, on examination of each of the three methodological points raised, we respectfully disagree that the original analyses require revision. In our view the analytic choices made in the published paper were justified, reported transparently and are supported by the Cochrane Handbook for Systematic Reviews of Interventions (version 6.5) [3]. We set out our reasoning below and, importantly, we note that the colleagues’ own re-analyses confirm the principal findings of the paper in two of the three outcomes they contest.

## Pooling of Measures Within the Dynamic Balance Meta-analysis

The commentary contends that including multiple directions of the Y-balance test (YBT) and left/right limb scores represents a unit-of-analysis error. We do not agree with that framing. Chapter 10 of the Cochrane Handbook (§10.3.2) explicitly permits the pooling of different scales or measures that capture the same underlying construct using the standardized mean difference (SMD), and the construct at stake here, dynamic postural control of the lower limbs, is well established in the sports-science and medicine literature as being expressed, variably, through composite YBT scores, through the anterior, posteromedial and posterolateral reach distances and through left and right limb scores. These measures are conceptually coherent with one another and represent different operationalization of the same construct rather than distinct outcomes.

Importantly, restricting inclusion to studies reporting composite YBT scores only would introduce a further source of bias, as computation of the composite score requires normalisation to limb length. In studies where only absolute reach distances are reported and limb length is not available, a composite score cannot be derived. Consequently, a substantial proportion of otherwise eligible studies would be excluded on purely reporting grounds rather than substantive methodological differences. The approach adopted in the original analysis therefore maximises data utilisation while maintaining conceptual consistency of the construct.

The YBT is a standardized derivative of the star excursion balance test (SEBT), developed to enhance measurement consistency and clinical applicability. It can be regarded as an instrumented and simplified version of the SEBT, reducing the assessment from eight reach directions to three. Empirical work has demonstrated strong associations between YBT and SEBT reach distances, supporting their use as closely related definitions of dynamic postural control [4, 5]. Accordingly, combining outcomes derived from these protocols within a single meta-analytic framework is consistent with their shared construct basis and established practice in the literature [6, 7].

We acknowledge that inclusion of multiple outcomes from the same study can, in theory, introduce statistical dependence. Given the conceptual alignment of these measures, the limited number of outcomes per study, and the consistency of findings in sensitivity analyses applying more restrictive outcome selection, we consider any such dependence unlikely to have influenced the direction or interpretation of the pooled effects across the outcomes examined. Furthermore, the inclusion of asymmetry data from Pardos-Mainer et al. [8] is justified, as their assessment followed the methodology of Gonzalo-Skok et al. [9], where balance and inter-limb asymmetry are evaluated as related components of neuromuscular control.

Additionally, two internal features of the original analysis support this position. First, the low heterogeneity observed (*I*^2^ = 7%) is consistent with these measures capturing a closely related underlying construct, although we recognise that *I*^2^ alone cannot establish measurement equivalence. Second, the random-effects model accounts for between-study differences in measurement, so the pooled estimate is a robust summary rather than a naive aggregation. The commentary’s own sensitivity analysis, restricted to studies that reported a YBT composite score, yielded a significant positive effect favouring FIFA 11 + (SMD = 0.70; 95% confidence interval [CI] 0.122–1.28; *p* = 0.018). This is consistent in direction and broadly comparable in magnitude to our original pooled estimate (SMD = 0.37; 95% CI 0.18–0.56; *p* < 0.001; *I*^2^ = 7%). The difference in magnitude is modest and well within the range expected given differences in outcome selection and precision. In other words, the colleagues’ preferred analysis and ours converge on the same substantive conclusion: FIFA 11 + improves dynamic balance in youth and adult soccer players. We therefore find no sufficient methodological basis to conclude that readers would be misled by the original analysis.

## Pooling of Measures Within the Change-of-Direction Speed Meta-analysis

The commentary raises the same concern regarding the inclusion of left and right limb values for the arrowhead agility test in Hwang and Kim [10] and of the T-test and the Illinois agility test in Nawed et al. [11]. We respectfully disagree in both cases. Change-of-direction (COD) speed is often treated as a single construct reflecting rapid deceleration, reorientation and acceleration. Field tests, such as the T-test, Illinois agility test and arrowhead test are widely used as interchangeable proxies in research and practice [12–14].

In line with this, the Cochrane Collaboration Handbook (Sect. 10.3.2) explicitly supports the aggregation of conceptually similar outcomes using the SMD framework when studies assess the same underlying construct with different measurement tools. Accordingly, the inclusion of multiple COD outcomes (e.g., left/right directions or different COD tests) from the same study is consistent with established meta-analytic practice, provided that they reflect the same performance domains. As noted above, the inclusion of multiple outcomes reflects different operationalization of the same construct rather than distinct domains.

We further note that the issue of dependence among effect sizes was examined directly. While methodological work has highlighted failure to account for within-study correlation as a potential source of bias in meta-analysis, particularly when large numbers of highly correlated outcomes are included [15], the magnitude of this issue is context-dependent. In the present analysis, no more than three dependent outcomes were included per study, and sample sizes were comparable across groups. To evaluate this empirically, we conducted a three-level meta-analysis (see Supplementary Material). These analyses demonstrated that within-study dependence contributed negligibly to the total variance for dynamic balance and vertical jump performance, and although some dependence was observed for COD, it did not affect the magnitude or statistical significance of the pooled estimates. Importantly, the results of the three-level models were consistent with those of the original analyses, reinforcing the robustness of the findings. Supplementary to this, numerous previous systematic reviews and meta-analyses have similarly combined multiple COD-related outcomes within a single analysis when assessing functionally comparable tests [16–18].

Decisively, the colleagues’ own sensitivity analysis conducted with the adjustments they advocate returned an estimate of SMD =  − 0.47 (95% CI − 0.78 to − 0.17; *p* = 0.002; *I*^2^ = 61.9%), which is statistically significant and fully consistent with the direction and magnitude of our original pooled estimate (SMD =  − 0.65; 95% CI − 1.11 to − 0.20; *p* = 0.005; *I*^2^ = 84%). The difference in magnitude is modest and does not alter the practical effect interpretation. Both analyses therefore support the same conclusion for practitioners.

## Pooling of Vertical-Jump Metrics

The commentary’s most substantive concern is with the inclusion of the countermovement jump (CMJ), Sargent jump, drop jump, squat jump and Bosco index within a single meta-analysis for vertical jump height. We have considered this argument carefully and, on reflection, maintain that the original pooling is defensible.

Each test included is, by design, a measure of vertical jump height as a proxy for lower-limb muscular power. Cochrane guidance explicitly supports combining such measures using the SMD when they capture the same conceptual domain. This approach is consistent with established practice in meta-analyses [6, 19, 20].

The commentary’s own jump-type-specific analyses yield point estimates that consistently favour FIFA 11 + (SMD = 0.28–0.32). These estimates fall within a consistent small-to-moderate range, albeit with some attenuation relative to the pooled estimate (SMD = 0.56). This attenuation is expected when analyses are disaggregated, as statistical power is reduced and confidence intervals widen.

Notably, the magnitude of these estimates clearly falls within a plausible range for training-induced adaptations. Methodological reviews have highlighted that implausibly large effect sizes (e.g., > 3 standard deviations) are frequently attributable to calculation errors rather than true effects [15]. The estimates observed here do not exhibit such characteristics, further supporting the validity of the pooled findings.

We acknowledge that each jump metric places different relative demands on the stretch–shortening cycle, on arm-swing coordination and on concentric versus reactive muscle action [21, 22]. This is precisely why heterogeneity was reported and why subgroup analyses were used to probe moderators. But the existence of mechanical differences between test modalities does not preclude their aggregation under a shared functional construct, any more than aggregating different pain scales or different measures of aerobic capacity would be precluded by their differing item content.

## On the Appropriate Use of Prediction Intervals

We share the colleagues’ enthusiasm for the reporting of 95% prediction intervals (PIs) alongside confidence intervals [23]. We note, however, that the PIs reported in the commentary’s sensitivity analyses (for example, − 1.59 to 2.99 for dynamic balance, − 1.44 to 0.50 for COD, − 0.91 to 1.53 for Sargent jump) reflect expected variability across contexts rather than absence of effect. They are therefore not grounds for dismissing the substantive conclusions.

## Summary

The commentary raises points of analytic preference rather than methodological error. Across all outcomes, sensitivity analyses, both those presented by the commentators and those conducted using multilevel models, yield consistent conclusions in direction, magnitude and interpretation.

We therefore maintain the published analyses and the interpretive conclusions that follow from them, including the subgroup analyses. We reaffirm that our approach is consistent with current methodological guidance and avoids several common statistical pitfalls identified in recent evaluations of meta-analytic practice in sport science [15]. For clarification, we conducted a three-level meta-analysis, and the corresponding results along with the R code have been provided in the Supplementary file to ensure transparency and reproducibility. We are grateful for the scholarly engagement with our work, and we remain open to continuing the methodological conversation in scientific literature.

## Supplementary Information

Below is the link to the electronic supplementary material.

Supplementary file1 (DOCX 24 KB)
