# Supplementary material for: Authors’ Reply to “Comment on: Effects of the FIFA 11+ Program on Physical Fitness in Youth and Adult Soccer Players: A Systematic Review and Meta-analysis”
Source: Sports Med. 2026 Jul 18;56(9):2345–8. doi: 10.1007/s40279-026-02488-3 (PMC13499820; doi:10.1007/s40279-026-02488-3)
Supplement: Supplementary file 1 [file 40279_2026_2488_MOESM1_ESM.docx]

**Supplementary Material**

**Authors’ Reply to “Comment on: Effects of the FIFA 11+ Program on Physical Fitness in Youth and Adult Soccer Players: A Systematic Review and Meta-analysis” (Manuscript SPOA-D-25-01955)**

Ibnu Noufal Kambitta Valappil^1^; Karuppasamy Govindasamy^2^; Gavoutamane Vasanthi^1^; Masilamani Elayaraja^1^; Cain C.T.Clark^3^; Koulla Parpa^4^; Borko Katanic^5^; Hüseyin Şahin Uysal^6^; [Hassane Zouhal](https://link.springer.com/article/10.1186/s40798-025-00831-y#auth-Hassane-Zouhal-Aff8-Aff9)^7,8^; Urs Granacher^9^**^*^**

1. Department of Physical Education and Sports, Pondicherry University, 605014 Puducherry, India
2. Department of Sports, Recreation and Wellness, Symbiosis International (Deemed University), Hyderabad Campus, Modallaguda (V), Nandigama (M), Rangareddy, 509217 Telangana, India
3. College of Life Sciences, Birmingham City University, B15 3TN, U.K.
4. Faculty of Sport and Exercise Science, UCLan University of Cyprus, Pyla 7080, Cyprus
5. Montenergin Sports Academy, Podgorica, Montenegro
6. Department of Physical Education and Sport, Faculty of Sport Sciences, Burdur Mehmet Akif Ersoy University, Burdur 15030, Turkey
7. M2S (Laboratoire Mouvement, Sport, Santé), Université Rennes, Rennes, France
8. Institut International des Sciences du Sport (2I2S), 35850, Irodouer, France
9. Department of Sport and Sport Science, Exercise and Human Movement Science, University of Freiburg, 79102 Freiburg, Germany

**Correspondence**

Name: Prof. Urs Granacher

Address: Department of Sport and Sport Science, Exercise and Human Movement Science, University of Freiburg, 79102 Freiburg, Germany

Email: [Urs.Granacher@sport.uni-freiburg.de](mailto:Urs.Granacher@sport.uni-freiburg.de)

<https://orcid.org/0000-0002-7095-813X>

**Sports Medicine**

**Supplementary Material 1**

**Dynamic Balance Overall effect size**

Multivariate Meta-Analysis Model (k = 15; method: REML)

Variance Components:

estim	sqrt nlvls fixed	factor

sigma^2.1 0.021 0.144	7	no	studyID

sigma^2.2 0.000 0.000	15	no studyID/effectsizeID

Test for Heterogeneity:

Q(df = 14) = 13.977, p-val = 0.451

Model Results:

estimate	se	zval	pval ci.lb ci.ub

0.388 0.108 3.581 <.001 0.176 0.601 ***

---

Signif. codes: 0 ‘***’ 0.001 ‘**’ 0.01 ‘*’ 0.05 ‘.’ 0.1 ‘ ’ 1

**Vertical Jump** Overall Effect Size

Multivariate Meta-Analysis Model (k = 15; method: REML)

Variance Components:

estim	sqrt nlvls fixed	factor

sigma^2.1 0.342 0.585	12	no	studyID

sigma^2.2 0.000 0.000	15	no studyID/effectsizeID

Test for Heterogeneity:

Q(df = 14) = 48.242, p-val < .001

Model Results:

estimate	se	zval	pval ci.lb ci.ub

0.563 0.195 2.885 0.004 0.181 0.946 **

---

Signif. codes: 0 ‘***’ 0.001 ‘**’ 0.01 ‘*’ 0.05 ‘.’ 0.1 ‘ ’ 1

**COD** Overall Effect Size

Multivariate Meta-Analysis Model (k = 14; method: REML)

Variance Components:

estim	sqrt nlvls fixed	factor

sigma^2.1 0.000 0.000	12	no	studyID

sigma^2.2 1.450 1.204	14	no studyID/effectsizeID

Test for Heterogeneity:

Q(df = 13) = 75.175, p-val < .001

Model Results:

estimate	se	zval	pval	ci.lb	ci.ub

-0.753 0.343 -2.197 0.028 -1.424 -0.081 *

---

Signif. codes: 0 ‘***’ 0.001 ‘**’ 0.01 ‘*’ 0.05 ‘.’ 0.1 ‘ ’ 1

**R Code for Three-level meta-analysis**

#effect size and its variance calculate

library(metafor)

effect_size <- escalc(measure="SMDH", m2i=control_mean, m1i=fifa_mean, sd2i=control_sd, sd1i=fifa_sd, n2i=control_n, n1i=fifa_n, data=fifa)

effect_size

\# overall effect size with three level meta analysis

'3 boyutlu meta analiz'

cod.ml <- rma.mv(yi, vi, random = ~ 1 | studyID/effectsizeID, data=effect_size)

print(cod.ml, digits=3)
